# Prostate cancer and PARP inhibitors: progress and challenges

**DOI:** 10.1186/s13045-021-01061-x

**Published:** 2021-03-29

**Authors:** Diego Teyssonneau, Henri Margot, Mathilde Cabart, Mylène Anonnay, Paul Sargos, Nam-Son Vuong, Isabelle Soubeyran, Nicolas Sevenet, Guilhem Roubaud

**Affiliations:** 1 Department of Medical Oncology, Institut Bergonie, Bordeaux, France; 2 Department of Genetic, Institut Bergonie, Bordeaux, France; 3 Department of Radiotherapy, Institut Bergonie, Bordeaux, France; 4 Department of Urology, Clinique Saint-Augustin, Bordeaux, France; 5 Department of Biopathology, Institut Bergonie, Bordeaux, France

**Keywords:** PARP inhibitors, Prostate cancers, Homologous recombination repair, DNA repair

## Abstract

Despite survival improvements achieved over the last two decades, prostate cancer remains lethal at the metastatic castration-resistant stage (mCRPC) and new therapeutic approaches are needed. Germinal and/or somatic alterations of DNA-damage response pathway genes are found in a substantial number of patients with advanced prostate cancers, mainly of poor prognosis. Such alterations induce a dependency for single strand break reparation through the poly(adenosine diphosphate-ribose) polymerase (PARP) system, providing the rationale to develop PARP inhibitors. In solid tumors, the first demonstration of an improvement in overall survival was provided by olaparib in patients with mCRPC harboring homologous recombination repair deficiencies. Although this represents a major milestone, a number of issues relating to PARP inhibitors remain. This timely review synthesizes and discusses the rationale and development of PARP inhibitors, biomarker-based approaches associated and the future challenges related to their prescription as well as patient pathways.

## Background

Prostate cancer is the second most common malignancy and the fifth cause of cancer death in men, worldwide [1]. Despite survival improvements achieved using next generation hormonal therapies, chemotherapies or radionuclides [2], prostate cancer remains lethal at the metastatic castration resistant stage (mCRPC). While androgen-receptor (AR) signaling still plays a central role in their development [3], a better understanding of the genomic landscape has highlighted that DNA-damage response (DDR) pathways may contribute to the progression of a large number of advanced prostate cancers [4, 5] often associated with worse prognosis [6].

DDR gene alteration induces a dependency on poly(adenosine diphosphate-ribose) polymerase (PARP)-1 for DNA repair, leading to cancer cell death when PARP-1 is inhibited. This synthetic lethality provides the rationale for using PARP inhibitors. Such treatments have already shown substantial survival benefits as maintenance therapy for patients with ovarian and pancreatic cancers or used in front line therapy in patients with breast cancers [7–9]. While several PARP inhibitors are still under development in prostate cancer, olaparib has just demonstrated an improvement in overall survival in patients with mCRPC harboring homologous recombination repair deficiency [10].

While this represents a major milestone to achieve, many issues remain unresolved regarding the efficacy of other PARP inhibitors, the potential toxicity of combinations, the panel of biomarkers to use, the mechanisms of resistance or the promising role of platinum salt-based chemotherapies. This review synthesizes and discusses the main points regarding the rationale and development of PARP inhibitors, the biomarker-based approaches associated and the future challenges related to their prescription as well as the patient’s pathway.

## From DNA-damage repair to the homologous recombination deficiency in advanced prostate cancer

### DNA-damage repair

DNA-maintenance machinery allows genome stability and prevents oncogenesis [11]. Single-strand breaks (e.g., insertion, deletion and mismatches) (SSBs) are repaired using base excision repair (BER), nucleotide excision repair (NER) or mismatch repair (MMR) processes. Those mechanisms are activated by various regulatory factors such as poly ADP-ribose polymerase (PARP), an ADP-ribosyl transferase, which contribute to recruit BER or NER proteins on the DNA strand break depending on the complexity of the point mutation.

PARP is a family of 17 distinct proteins, in which PARP 1 and 2 are involved in DNA repair [12]. PARP1 binds to damaged DNA gaps and, after conformational change, induces PARylation [13]. PARP1 catalyzes the polymerization of ADP-ribose units from NAD + (nicotinamide adenine dinucleotide) molecules and recruits proteins of the DNA SSB repair system (BER or NER systems) [14, 15]. Once a repair complex is recruited to SSB, PARP1 is released from DNA.

When PARP1 is deficient, SSB cannot be repaired [16]. Through the process of DNA replication, SSB converts into a double-strand breaks (DSB), and the DSB repair system is then required. Three mechanisms exist to repair DSB: non-homologous end joining (NHEJ), microhomology-mediated end joining (MMEJ) and homologous recombination repair (HRR) [17]. HRR is the most conservative of them, leading to a high-fidelity repair by avoiding loss of information with the use of the second chromosome as a template.

### Homologous recombination repair (Fig. 1)

**Fig. 1:**
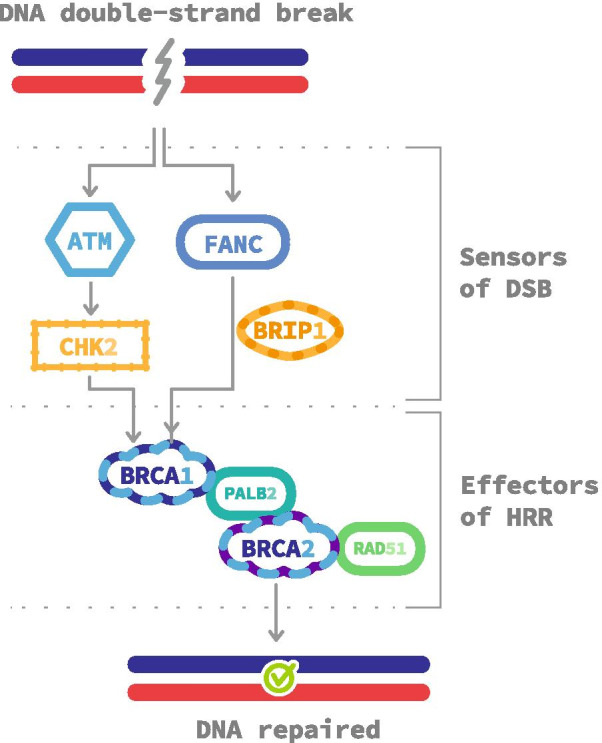
Homologous recombination repair pathways. Double-strand breaks are detected by different proteins such as FANC complex or ATM/CHEK2. They recruit homologous stand invasion effectors, including BRCA1, BRCA2, PALB2 and RAD51, on the break site to carry out a faithful DNA repair. *DSB* double-strand break, *HRR* homologous recombination repair

Following a DSB, ATM, a serine/threonine kinase, is recruited and activated at the site. ATM activates, via CHK2, a phosphorylation cascade of several targets including BRCA1 [18]. BRCA1 recruits PALB2 which attracts BRCA2 and RAD51 [19, 20]. This complex induces the transfer of the broken sequence to the respective undamaged sequence of the second chromosome, allowing DNA restoration through a complex cascade of many protein interactions. This core process is called homologous pairing by strand invasion [21]. BRCA1 can also be activated through the Fanconi Anemia (FANC) and BRIP1 pathway [22].

### Homologous recombination deficiency in metastatic castration-resistant prostate cancer: prognostic impact of germinal mutations

Ninety percent of mCRPC carry at least one clinically actionable molecular alteration, with androgen receptor (AR) pathways being the most frequently altered (71%) [4]. Approximately 27% of mCRPC have a germline or a somatic alteration in *BRCA2*, *BRCA1*, *ATM* or *CHEK2* [5]. All HRR genes are tumor suppressors and require both alleles to be inactivated in the tumoral cell, with a complete loss of protein expression, according to the Knudson two-hit hypothesis. The first allele could either be constitutively or somatically inactivated. The second hit could be due to another somatic event, which leads to a complete loss of HRR system within the cell. A copy neutral loss of heterozygosity (LOH) with the loss of the wild-type allele is the most common phenomenon. When tumors are diagnosed with only one germline alteration without any other mutant allele (either by somatic mutation or LOH), HRR is still working and is not considered as a driver of oncogenesis [23]. Taking into account both somatic and germline mutations, *BRCA2* (12–18%), *ATM* (3–6%), *CHEK2* (2–5%) and *BRCA1* (< 2%) are the most common altered genes involved in HRR [4, 5].

In a cohort of patients with mCRPC, 11.8% presented with a germline mutation in HRR genes, with an associated LOH in more than 90% of cases [24]. Interestingly, this cohort did not reveal any association with age and familial history of cancer, although most patients were younger than 70 years old [24]. These germline mutations represent roughly half of the cases of homologous recombination deficient (HRD) mCRPC [5]. *BRCA2* alterations are still the most frequent, responsible for 5.3% of cases, followed by *ATM*, *CHEK2* and *BRCA1* (1.9, 1.6 and 0.9%, respectively) [24].

Germline mutations of *BRCA1/2* and *ATM* are associated with worse prognosis in prostate cancer, while to-date, somatic mutations are not shown to be [6, 25]. Patients with germline *BRCA2* pathogenic variants have a 20-fold risk of death related to prostate cancer [26]. *ATM,* a DNA-damage sensor gene, is frequently altered in mCRPC. While a complete inactivation of *BRCA2*, *BRCA1* and *PALB2* imprints a distinct mutational signature on genomes, there is no such a pattern with *ATM* biallelic inactivation [27]. *CHEK2* is a checkpoint kinase gene, activated by *ATM* and regulating *BRCA1*. The most common pathogenic *CHEK2* mutation, c.1100delC, has been associated with an increased risk of lethal *versus* indolent prostate cancers (respectively, 1.28% vs. 0.16% *P* = 0.004), giving a poor prognosis to this mutation [28]. Minor genes such as *BRIP1, RAD51D* or *PALB2* are found in less than 0.5% of cases [29, 30].

Deleterious *CDK12* alterations are enriched from localized to advanced prostate cancers, occurring in 5–11% of patients with mCRPC, and associated with worse prognosis and high Gleason scores [31–33]. CDK12 is known to inhibit intronic polyadenylation sites (frequent in HRR genes sites), in order to keep the last exons within the transcript and avoid truncated proteins. While correlated to low expression of HRR genes, bi allelic *CDK12* inactivation was more recently shown to be associated with a distinct subgroup of prostate cancers characterized by focal tandem duplications and gene fusion-induced neoantigens all over the genome, as well as CD4 + FOXP3—tumor-invasive lymphocytes (i.e., likely to be permissive) [28, 33]. In line with these pre-clinical data, a retrospective study suggested a better sensitivity of *CDK12* alterations to immune checkpoint inhibitors [31].

Alterations in HRR genes are present in a significant proportion of patients with mCRPC, but their clinical implication remains unclear. Recently, Castro et al*.* investigated the impact of germline mutations in HRR genes in the prospective PROREPAIR-B study [34]. DDR alterations were identified in 68 of 419 patients with mCRPC (16.2%), including 14 with *BRCA2*, 8 with *ATM*, 4 with *BRCA1* and none with *PALB2* or *CHEK2* mutations. The results did not show a difference in cause-specific survival (CSS) between altered and non-altered mCRPC (*P* = 0.65). Conversely, CSS for patients with *BRCA2* alterations was approximately halved compared to non-carriers (17.4 months vs. 33.2 months, 95% CI 1.07–4.10, *P* = 0.27). The authors conducted a post hoc analysis to compare the CSS and PFS2 (second progression or death) according to *BRCA2* status and sequence: taxane-new hormonal therapy (NHT) or NHT-taxane. After multivariate analysis it seemed that g*BRCA2* carriers had a shorter CSS and PFS2 compared to non-carriers in the taxane-NHT sequence, while no difference was observed in the NHT-taxane sequence. This suggests that g*BRCA2* alterations are correlated with poorer prognosis, but that NHT may reverse this effect. These results are reinforced by the retrospective study of Antonarakis et al*.,* showing better progression-free survival (PFS) and overall survival (OS) in patients with g*BRCA*/*ATM* alterations treated with NHT as first line, compared to non-mutated patients. The outcomes after first line NHT did not appear better for patients with non-*BRCA*/*ATM* germline alterations [35]. The results of these two studies need to be confirmed with larger groups of patients. Similar studies regarding somatic HRR alterations were not found.

Overall, a growing body of evidence showing a poorer prognosis associated with germinal HRR gene aberrations as well as mitigated results of standard treatments in patients with prostate cancer, strengthened the rationale to develop specific treatments such as PARP inhibitors in this setting.

## Development of PARP inhibitors in prostate cancer used alone or in combination

### Mechanism

**Fig. 2:**
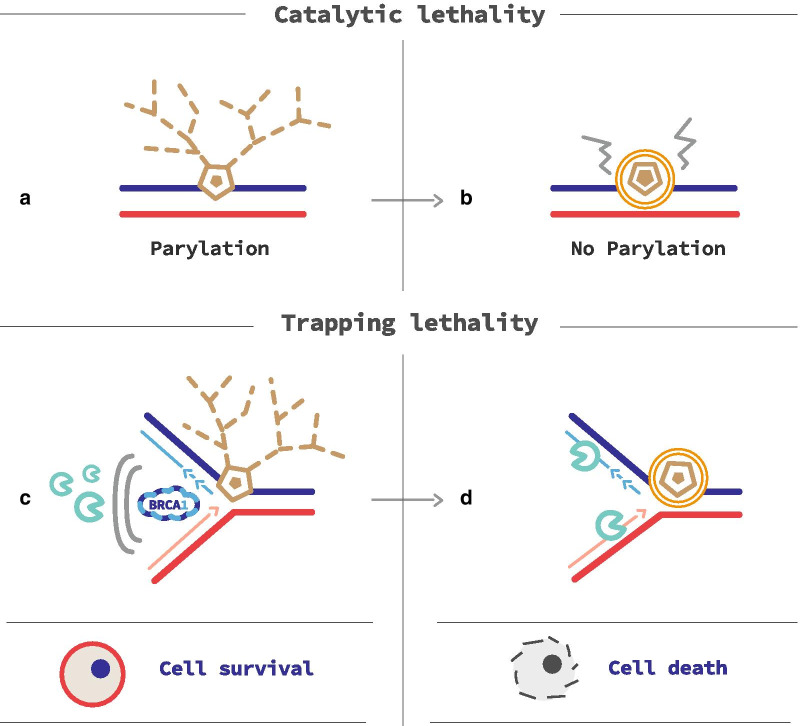
PARP inhibitors action mechanisms. Catalytic lethality. **a** PARP is recruited on single-stand breaks (SSB) and PARylates to recruit base-excision repair (BER) agents to repair SSB. **b** PARPi are competitive inhibitors of PARP and prevent PARylation from occurring. So, BER systems are not recruited and SSB is not repaired, allowing synthetic lethality. Trapping lethality. **c** Nascent DNA on replication forks is protected from nuclease action by a BRCA1/2 shield. **d** Inactivated PARP is locked on SSB; thus, the replication fork gets stale. In BRCA1/2 deficient cells, nucleases have the time to degrade nascent DNA, leading to cell death

**Fig. 3:**
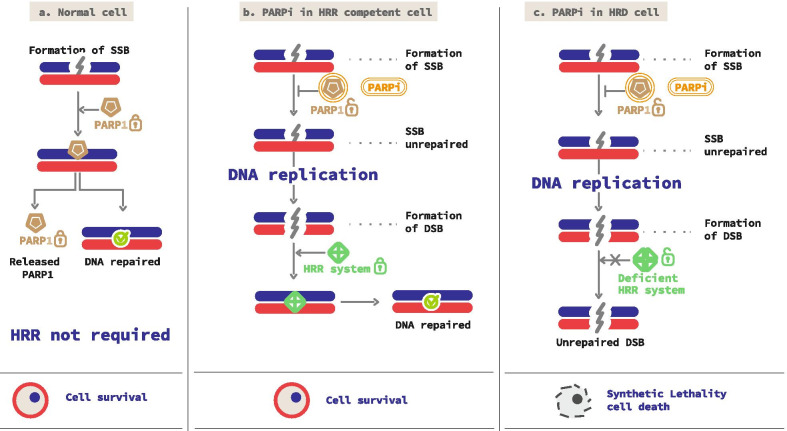
Principle of synthetic lethality reached with PARP inhibitors (PARPi) used in homologous recombination deficient (HRD) cells. **a** In standard conditions, PARP proteins repair single-stand breaks (SSB). **b** In homologous recombination repair (HRR)-competent cells, the use of PARPi prevents SSB from repairing. Though replication, this converts SSB into double-strand breaks (DSB), and cells survive using HRR. **c** In HRD cells with PARPi, neither SSB nor DSB could be repaired. This leads to cell death, through a synergy between PARPi and HRD called synthetic lethality

PARP inhibitors (PARPi) are oral-targeted therapies, which competitively bind to the NAD + sites of PARP1 and PARP2 inducing a catalytic inhibition (Fig. 2a, b). Five different molecules are currently under development or recently approved: olaparib, rucaparib, niraparib, veliparib and talazoparib. Their action inhibits the PARylation, and thus, SSBs repair, leading to increase the number of SSB within the cell (Fig. 3a). Unrepaired SSB converts into DBS during the replication (Fig. 3b). When used on HRD cells, PARPi induce a synergistic lethal effect, increasing genomic instability enough to reach tumor cell death. This phenomenon is called synthetic lethality and is specific to tumor cells with complete HRD (Fig. 2c). Catalytic inhibition has been observed in prostate cancer cell lines [36, 37].

PARPi also trap PARP enzymes on DNA. The resulting DNA-PARP complexes block the replisome during replication, in a way that only HRR can resolve (Fig. 2c, d) [36]. BRCA1, BRCA2 and PALB2 are not only involved in HRR, but also stabilize stalled replication forks. BRCA1/2 or PALB2 loss destabilizes replication forks, and this effect is potentialized by PARPi trapping, in a synthetic lethality process [38]. Thus, PARPi trapping has a cytotoxic action in HRD cells, and more accurately on BRCA1, BRCA2 or PALB2 deficient cells. Potency of trapping varies according to the molecule of PARPi used (e.g., major for talazoparib) [36, 37].

### PARP inhibitors as single agents

According to the preclinical rationale described above, PARPi were firstly developed as single agents in mCRPC. The main clinical studies are summarized in Table 1.

**Table 1:** Phase II or III trials using PARP inhibitors alone to treat prostate cancers

CTID	Treatment	Phase	No. patients or estimated enrollment	Disease status	Mandatory HRR status for inclusion	Determination method for HRD	Primary endpoints	Results
NCT01682772/TOPARP-A	Olaparib	2	50	mCRPC after at least docetaxel	No	Tumor	Composite response rate^b^	All comers: 33% HRD: 88%
NCT01682772/TOPARP-B	Olaparib	2	98	mCRPC after at least docetaxel	Bi-allelic deleterious HRD	Tumor	Composite response rate^b^ Preplanned secondary endpoint: ORR	*BRCA1/2*: 83%, ORR: 52.4% *PALB2*: 57%, ORR: 33.3% *ATM*: 37%, ORR: 8.3% *CDK12*: 25%, ORR: 0%
NCT02987543/PROfound	Olaparib versus NHT	3	778	mCRPC after at least 1 NHT	Bi- or mono-allelic somatic or germline deleterious HRD	Tumor	Radiographic PFS Preplanned secondary endpoint: OS	rPFS: *BRCA/ATM*: 7.4 months versus 3.6mo, HR = 0.34 (95% CI 0.25–0.47) General HRD: 5.8 months versus 3.5 months, HR = 0.49 (95% CI 0.38–0.63) OS: *BRCA/ATM*: 19.1 months versus 14.7 months HR = 0.69 (CI 95% 0.5–0.97) No-*BRCA/ATM*: 14.1 months versus 11.5 months HR = 0.96 (CI 95% 0.63–1.49)
NCT03432897/BrUOG-337	Olaparib	2	13	High-risk localized PC	Deleterious HRD^a^	Tumor or plasma	PSA response rate prior prostatectomy	Recruiting
NCT03047135	Olaparib	2	50	Castration Sensitive Biochemically Recurrent nmPC	No	Undescribed method	PSA response rate	Recruiting
NCT03434158 /IMANOL	Olaparib as maintenance therapy after docetaxel	2	27	mCRPC after at least docetaxel	Deleterious HRD^a^	Undescribed method	Radiographic PFS	Recruiting
NCT03012321/BRCAAway	Olaparib versus abiraterone versus abiraterone + olaparib	2	70	mCRPC, 1st line	No	Tumor	PFS	Recruiting
NCT03263650	Olaparib maintenance after cabazitaxel–carboplatin combination	2	123	Aggressive variant PC	No	Not performed	PFS	Ongoing, not recruiting
NCT02854436/GALAHAD	Niraparib	2	291	mCRPC after at least 1 chemotherapy and 1 NHT	Bi-allelic HRD or germline pathogenic BRCA1/2 alteration^b^	Tumor or plasma	ORR	*BRCA*: 41% Non-*BRCA*: 9%
NCT04288687	Niraparib	2	18	mCRPC, platine sensitivity	Deleterious HRD^a^	Undescribed method	Radiographic PFS	Not yet recruiting
NCT04037254	ADT (24 months) + RT + niraparib (12 months)	2	180	High-risk localized PC	No	Not performed	Disease-free survival	Ongoing, not recruiting
NCT04030559	Niraparib for 3 months	2	30	High-risk localized PC, prior prostatectomy	Bi- or mono-allelic deleterious HRD	Undescribed method	Pathologic response rate	Recruiting
NCT02952534/TRITON-2	Rucaparib	2	193	mCRPC after at least 1 chemotherapy and 1 NHT	Bi- or mono-allelic somatic or germline deleterious HRD	Tumor or plasma	ORR and PSA response rate (PRR)	s*BRCA1/2*: 43.9%, PRR: 50.7% g*BRCA1/2*: 42.9%, PRR: 61.4% *ATM*: 10.5%, PRR: 4.1% *CDK12*: 0%, PRR: 6.7% *CHEK12:* 11.1%, PRR: 16.7%
NCT02975934/TRITON-3	Rucaparib versus NHT or docetaxel	3	400	mCRPC, after 1 NHT, 0 chemotherapy	Deleterious BRCA1/2 or ATM alteration^a^	Undescribed method	Radiographic PFS	Recruiting
NCT03413995/TRIUMPH	Rucaparib	2	30	mCSPC unfit/unwilling ADT	Germline HRD alteration^b^	Undescribed method	PSA response rate	Recruiting
NCT03442556/PLATI-PARP	Rucaparib maintenance after docetaxel–carboplatin combination	2	20	mCRPC	Bi- or mono-allelic deleterious HRD	Tumor or plasma	Radiographic PFS	Recruiting
NCT03533946/ROAR	Rucaparib	2	32	nmCSPC	Deleterious HRD^a^	Tumor or plasma	PSA response rate	Recruiting
NCT03148795/TALAPRO-1	Talazoparib	2	100	mCRPC after at least 1 chemotherapy and 1 NHT	Mono- or bi-allelic HRD (*CDK12* excluded)	Tumor	ORR	*BRCA*: 50% *ATM*: 7% Other HRD: 0%

*ATM*: ataxia telangiectasia mutated, s*BRCA*: somatic deleterious mutation of *BRCA*. g*BRCA*: germinal deleterious mutation of *BRCA*, *mCRPC* metastatic castration-resistant prostate cancer, *mCSPC* metastatic castration-sensitive prostate cancer, *nmPC* non-metastatic prostate cancer, *nmCSPC* non-metastatic castration-sensitive prostate cancer, *ADT* androgen deprivation therapy, *NHT* new hormonal therapy, *HRR* homologous recombination repair, *HRD* homologous repair deficiency, *CTID* clinical trial identification, *PC* prostate cancer, *PFS* progression free survival, *ORR* objective response rate, PSA response rate: decline of more than 50%, *RT* radiotherapy

^a^Mono- or bi-allelic status not specified

^b^Germline or somatic alteration specified

^c^Composite response rate: response according to RECIST or PSA reduction > 50%, or reduction of circulating tumor cells to less than 5/7.5 mL of blood confirmed 4 weeks later

#### Olaparib

Olaparib was the first PARPi to be described. After promising results reported in phase I, fifty patients with mCRPC were enrolled in a phase II study (TOPARP-A) [39]. They had received at least one chemotherapy regimen, and 98% were previously treated with abiraterone or enzalutamide. The primary *endpoint* was the composite response rate (CRR), defined as an objective radiological response (ORR) based on the response evaluation criteria in solid tumors (RECIST), or a reduction of at least 50% of the PSA level, or a decrease in the circulating tumor cell (CTC). Of the 49 patients assessed for CRR, seven had *BRCA2* and four had *ATM* alterations. Other HRR aberrations were seen in *BRCA1*, *CHEK2*, *FANCA*, *HDAC2* or *PALB2* genes. Fourteen of the 16 patients (88%) with HRR alterations had a CRR compared to 6% for the patients without HRR deficiencies. Olaparib was well tolerated; grade 3 or 4 adverse events were primarily anemia (20%), fatigue (12%) and leukopenia (6%). Using next-generation sequencing (NGS), TOPARP-B selected 161 (27%) patients with HRR gene alterations among 592 patients with mCRPC treated with at least one chemotherapy, and eligible for NGS analysis, [40]. Given the dose/response relationship for olaparib [41, 42], ninety-eight patients were randomly assigned in 2 cohorts: one receiving olaparib 400 mg twice daily and the other 300 mg twice daily. The primary endpoint was CRR defined with the same criteria as TOPARP-A. A confirmed CRR was observed in 54.3% of the patients in the 400 mg arm, compared to 39.1% in the 300 mg cohort with, in return, higher toxicity in the 400 mg arm (3 times more patients requested a dose reduction in the arm 400 mg mostly due to anemia). A preplanned subgroup analysis showed that the highest CRR was observed in the *BRCA1*/*2* subgroup (83.3%), then *PALB2* (57.1%), both effectors of the HRR system. ORR was 52.4% and 33.3% for *BRCA1/2* and *PALB2* subgroups, respectively. The CRR was lower in the *ATM* and *CDK12* subgroups (36.8% and 25.0%, respectively), and moreover, almost no radiological or PSA responses were observed. The effect of olaparib in the *CDK12* subgroup might be influenced by an imbalance with more alterations in groups with lower doses. While radiological and biological responses are commonly used in daily practice, the value of CTC conversion as a biomarker of response is still under evaluation, even if, in this trial, CTC conversion was associated with better radiographic PFS (rPFS) and OS. Thus, the CRR for the *ATM* and *CDK12* subgroups should be interpreted with caution.

TOPARP studies confirmed that Olaparib used alone was more efficient in HRR deficient mCRPC. Moreover, the efficacy in terms of CRR seemed to be higher for effector genes of the HRR system (*BRCA, PALB2*) than sensors (*ATM, CDK12*)*.*

Building on these results, the PROfound study was designed [43]. This phase III trial compared olaparib to NHT in patients with mCRPC progressing after at least one treatment with enzalutamide or abiraterone. Previous taxane chemotherapy was allowed. Patients were included if an alteration (mono- or bi-allelic) in at least 1 of the 15 prespecified HRR genes (*BRCA1, BRCA2, ATM, BRIP1, BARD1, CDK12, CHEK1, CHEK2, FANCL, PALB2, PPP2R2A, RAD51B, RAD51C, RAD51D,* and *RAD54L*) was found. Patients with an alteration in *BRCA1, BRCA2* or *ATM* were assigned to cohort A, and patients with alterations in any of the 12 other genes were allocated to cohort B. The primary endpoint was the imaging-based PFS in cohort A. If the primary objective was reached, testing of key secondary *endpoint*s had to be performed in a hierarchical manner: ORR in cohort A, rPFS in the overall population, time to pain progression in cohort A and OS in cohort A. Crossover to olaparib was allowed after blinding central review imaging-based progression. After analysis of 2792 samples, 778 (28%) patients were randomly assigned in a 2:1 ratio to receive olaparib at a dose of 300 mg twice daily (162 patients in cohort A, 94 patients in cohort B) or the physician’s choice between enzalutamide or abiraterone (83 patients in cohort A, 48 patients in cohort B). Primary endpoint was reached and the median imaging-based PFS in the cohort A was longer in the olaparib group (7.4 months vs. 3.6 months, Hazard Ratio (HR) 0.34, 95% CI 0.25–0.47, *P* < 0.001). The ORR, assessed by blinded, independent, central review, among evaluable patients of the cohort A was 33% in the olaparib group and 2% in the control group. PFS was also longer in the overall population (cohort A and B) for the experimental group (5.8 months) compared to the control group (3.5 months), *P* < 0.001. A statistically meaningful better OS was subsequently reported for patients receiving olaparib in cohort A than those treated with NHT (19.1 months vs. 14.7 months, HR 0.69, 95% CI 0.50–0.97, *P* = 0.0175) [10]. Among patients in the control group, with imaging-based disease progression, 81% crossed over to receive olaparib, and the sensitivity analysis adjusting for the crossover showed an HR of 0.42 (95% CI 0.19–0.91). No difference was observed before or after adjustment for crossover (HR 0.96; 95% CI 0.63–1.49) for cohort B.

The PROfound study is the first trial showing an improvement in OS for mCRPC with an alteration in *BRCA1*/*2* or *ATM* genes, treated with PARPi, after at least the use of NHT, even if a substantial crossover was observed. In addition, olaparib significantly improved time to pain progression, a key secondary endpoint (HR 0.44; 95% CI 0.22–0.91, *P* = 0.0192), and was associated with better health-related quality of life (HRQoL) functioning over time, compared with NHT [44, 45]. These patient-reported outcomes are of importance in the setting of advanced prostate cancer. However, even if promising CRR rates were reported by TOPARP studies as well as rPFS regarding *ATM* altered patients, the inclusion in the same cohort of *ATM* and *BRCA* is questionable given function differences and marginal results in other phase III studies. A large retrospective study [46] and the CARD randomized phase III trial [47] showed that not delaying chemotherapy for eligible patients improves survival. Given these results, a standard arm allowing docetaxel may have been more appropriate. Indeed, 35% of the patients allocated to the standard arm received two consecutive NHT, but no taxane-based chemotherapy, making the control group weaker. However, the PROfound trial was designed prior to these retrospective and CARD data [46, 47]. Other ongoing phase II studies, evaluating olaparib as maintenance therapy following chemotherapy for mCRPC, or as a single agent for localized prostate cancers, are reported in Table 1.

#### Niraparib

Niraparib is another PARP1/2 inhibitor with higher trapping potency and cytotoxicity than olaparib [36]. GALAHAD is an ongoing open-label phase II study evaluating niraparib in patients with mCRPC and HRR deficiency, progressing after taxane and NHT. HRR deficiency was defined as a biallelic alteration in *BRCA1/2, ATM, FANCA, PALB2, CHEK2, BRIP1*, or *HDAC2* assessed by a plasma or tissue-based test. Patients received 300 mg of niraparib daily. The primary endpoint was the ORR. A CRR, defined as ORR, conversion of CTC, or more than 50% decline in PSA level, was evaluated. Preliminary results were recently reported, regarding a population of 81 patients with a biallelic HRR deficiency (46 *BRCA1/2* and 35 non-*BRCA*) [48]. Of the 51 patients with a measurable disease, the ORR in the “*BRCA* group” was 41% (95% CI 23.5–61.1%) compared to 9% (95% CI 1.1–29.2%) in the “non-*BRCA* group”; and the CRR was 63% (95% CI 47.6–76.8%) compared to 17% (95% CI 6.6–33.7%), respectively. Median PFS and OS in *BRCA* were 8.2 months (95% CI 5.2–11.1 months) and 12.6 months (95% CI 9.2–15.7 months), respectively, versus 5.3 months (95% CI 1.9–5.7 months) and 14.0 months (95% CI 5.3–20.1 months) in non-*BRCA*. Grade 3 and 4 adverse events were mostly hematologic with anemia (29%), thrombocytopenia (15%) and neutropenia (7%), managed with dose reduction or interruption.

GALAHAD is a phase II study with a small number of patients. Thus, results on efficacy need to be interpreted cautiously. As for olaparib, niraparib seems to be more efficient in *BRCA-*altered patients. Efficacy is in the same order of magnitude as olaparib (ORR of 52.4% and 41% for *BRCA* groups in TOPARP-B and GALAHAD, respectively) even if we would expect a greater effect in a pure biallelic population. Toxicity was manageable and similar to olaparib.

Three other ongoing phase II studies evaluating niraparib as monotherapy are reported in Table 1. One for mCRPC and two for localized prostate cancer. To date, there is no phase III study investigating niraparib as monotherapy.

#### Rucaparib

Rucaparib has a cytotoxic power and trapping efficacy comparable to olaparib [37]. The TRITON-2 phase 2 trial evaluated rucaparib 600 mg twice daily in patients with mCRPC and mono- or bi-allelic deleterious somatic or germline alteration in HRR genes (*BRCA1, BRCA2, ATM, BARD1, BRIP1, CDK12, CHEK2, FANCA, NBN, PALB2, RAD51, RAD51B, RAD51C, RAD51D*, or *RAD54L*). Alterations were tested on plasma or tumor tissue. Patients had a progression after one or two NHT and one taxane-based chemotherapy. Primary outcomes were the ORR and the PSA response rate. One hundred and fifteen patients had a *BRCA1/2* alteration (44 germline, 71 somatic) [49]. The ORR was 43.5% (95%CI 38.1- 63.4%) for the 62 ORR-evaluable patients; no differences were observed between germline and somatic mutated patients. With a median follow-up of 17.1 months, the median duration of response was not reached yet. The PSA response rate was 54.8% (95% CI 45.2–64.1%). PSA responses seemed smaller in the *BRCA1* (15.4%; 2 of 13 patients) or mono-allelic patients (11.1%; 1 of 9 patients) compared to *BRCA2* (59.8%; 61 of 102 patients) or biallelic patients (75.0%; 27 of 36 patients) (although these are small populations). Seventy-eight patients had a non-BRCA HRR alteration [50]. The PSA response rate was 4.1%, 6.7% and 16.7% in the *ATM* group (49 patients), *CDK12* cohort (15 patients) and *CHEK2* group (12 patients), respectively. The ORR was 10.5% in the *ATM* group (19 evaluable patients), 0% in the *CDK12* cohort (10 evaluable patients), and 11.1% in the *CHEK2* group (9 evaluable patients). Of the 14 evaluable patients with other HRR gene alterations, 4 (28.6%) had a radiographic response and 5 (35.7%) had a PSA response. Encouraging results are seen for *PALB2*, *BRIP1* and *RAD51.* Indeed, both patients with a *PALB2* alteration had PSA responses and one had a partial radiographic response. One of the 2 *BRIP1*-altered patients and a patient with an *RAD51B* alteration had radiographic and PSA responses, both ongoing [50]. Adverse events were comparable to other PARPi.

Again, these results suggest that *BRCA1/2* and *PALB2,* both effectors of HRR, are interesting targets, while *ATM, CDK12* and *CHEK2,* sensors of HRR, may be less directly involved. The other non-BRCA HRR genes warrant further investigation. This is the first phase II study comparing mono- and biallelic, somatic and germline altered patients. The results, even with small cohort of patients, raise hypotheses for further investigations suggesting better efficacy for biallelic altered patients and no differences between somatic and germline status.

The TRITON-3 study (NCT02975934) is an ongoing phase III trial comparing rucaparib *versus* abiraterone, enzalutamide or docetaxel (physician’s choice) after 1 NHT but no chemotherapy, for patients with mCRPC and a deleterious mutation of *BRCA1/2* or *ATM* (Table 1).

#### Talazoparib

Talazoparib is the PARPi with the strongest trapping efficiency [37]. Its efficacy is 20- to 200-fold greater than the others [51]. TALAPRO-1 is an ongoing phase II trial evaluating talazoparib 1 mg daily in patients with mCRPC and mono- or bi-allelic HRR gene alterations (*CDK12* was not considered as an HRR gene). They have received at least one taxane-based chemotherapy regimen and one or more NHT. Interim results were recently reported with 113 patients having received talazoparib and 75 patients evaluable for the primary endpoint of ORR (41 *BRCA1/2*, 3 *PALB2*, 17 *ATM* and 14 patients with other HRR gene alterations) [52, 53]. In the *BRCA, PALB2* and the *ATM* groups, ORR was 43.9%, 33.3% and 11.8%, respectively. No response was observed in the other HRR groups. Most common adverse events were slightly more frequent than other PARPi, namely anemia (42.5%, all grades) and nausea (32.7%, all grades) [53].

With these convincing results, PARPi are becoming accepted as options to treat patients with mCRPC. *BRCA1/2* alterations are predictive of response to PARPi and are the best candidates for treatment, irrespective of somatic or germinal status. The biallelic inactivation also appears to be important, with most of the responses observed in this group. More studies are awaited to support these statements and will not only explore the PARPi efficacy earlier in the medical care (i.e., mCSPC or localized prostate cancers) but also in combination with other drugs.

### Treatment combination with PARP inhibitors

Even if sustained efficacy is observed with PARPi used alone, primary and secondary resistances are seen. To try to potentiate their action, trials are underway to evaluate the use of PARPi in combination with other drugs. The main studies are summarized in Table 2.

**Table 2:** Phase II or III trials using PARP inhibitors in combination to treat prostate cancers

CTID	Treatment	Phase	No. patients or estimated enrollment	Disease status	Mandatory HRR status for inclusion	Determination method for HRD	Primary endpoints	Results
NCT01972217	Abiraterone ± olaparib	2	142	mCRPC after docetaxel, no prior NHT	No	Plasma or blood or tumor	Radiographic PFS	13.8 months (combination) versus 8.2 months (control), *P* = 0.034, HR = 0.65 (CI 95% 0.44–0.97)
NCT03732820/PROpel	Abiraterone ± olaparib	3	720	mCRPC, 1st line	No	Tumor	Radiographic PFS	Ongoing, not recruiting
NCT02484404	Olaparib + durvalumab	2	17	mCRPC after 1 NHT	No	Not performed	Clinical efficacy	PSA response rate: 53%, Radiographic response: 44% Radiographic PFS: 16.1 months
NCT02861573/KEYNOTE-365 cohort A	Olaparib + pembrolizumab	2	41	mCRPC after at least docetaxel	No	Not performed	PSA response rate, safety	PSA response rate: 13%
NCT03834519/KEYLINK-010	Olaparib + pembrolizumab vs NHT	3	780	mCRPC after CT and 1 NHT	No	Not performed	OS, radiographic PFS	Recruiting
NCT03810105	Olaparib + durvalumab	2	32	Castration Sensitive Biochemically Recurrent nmPC	Bi- or mono-allelic deleterious HRD	Undescribed method	Number of undetectable PSA	Recruiting
NCT04336943	Olaparib + durvalumab	2	30	Castration Sensitive Biochemically Recurrent nmPC	Deleterious HRD, bi-allelic *CDK12* alteration, MSI^a^	Undescribed method	Number of undetectable PSA	Recruiting
NCT03787680 /TRAP	Olaparib + ATRi (AZD6738)	2	45	mCRPC after docetaxel or 1 NHT	Cohort 1: no Cohort 2:^a^ HRD	Tumor or blood	ORR in DNA repair proficient patients	Recruiting
NCT03516812	Olaparib + testosterone	2	30	mCRPC after 1 NHT, no chemotherapy	50% with^a^ deleterious HRD, 50% with competent HRR	Tumor or blood	PSA decrease, AES	Recruiting
NCT02893917	Olaparib ± cediranib	2	90	mCRPC after 2 lines of treatment	No	Not performed	Radiographic PSA	Ongoing, not recruiting
NCT03748641/MAGNITUDE	Abiraterone ± niraparib	3	1000	mCRPC, 1st line	No	Tumor	Radiographic PFS	Recruiting
NCT04497844/AMPLITUDE	ADT + abiraterone ± niraparib	3	788	mCSPC	No	Tumor	Radiographic PFS	Recruiting
NCT03431350	Niraparib + cetrelimab or niraparib + abiraterone	1–2	148	mCRPC after 1 or 2 NHT	No	Not performed	ORR and AES	Recruiting
NCT04455750/CASPAR	Enzalutamide ± rucaparib	3	1002	mCRPC, 1st line	No	Tumor	Radiographic PFS and OS	Not yet recruiting
NCT03338790/CheckMate 9KD	Nivolumab + rucaparib/enzalutamide/docetaxel	2	330	mCRPC	No	Not performed	ORR and PSA response rate	Ongoing, not recruiting
NCT03395197 (TALAPRO-2)	Enzalutamide ± talazoparib	3	1037	mCRPC, 1st line	No	Not performed	Radiographic PFS	Recruiting
NCT04332744/ZZ-first	ADT + enzalutamide ± talazoparib	2	54	mCSPC high volume	No		PSA complete response rate	Recruiting

*CRPC* metastatic castration-resistant prostate cancer, *mCSPC* metastatic castration-sensitive prostate cancer, *nmPC* non-metastatic prostate cancer, *NHT* new hormonal therapy, *HRR* homologous recombination repair, *HRD* homologous repair deficiency, *CTID* clinical trial identification, *PFS* progression free survival, *ORR* objective response rate, *OS* overall survival, PSA response rate: decline of more than 50, *AES* adverse events. ATRi: ceralasertib: orally available inhibitor of ataxia telangiectasia and rad3 related (ATR) kinase

^a^Mono- or bi-allelic status not specified

#### PARP inhibitors and new hormonal therapies

In 2013, Polkinghorn et al*.* showed that AR regulates a transcriptional program of DNA repair genes and that NHT results in downregulation of DNA repair genes [54]. Moreover, Schiewer et al*.* demonstrated, using different cell lines and xenografts, that PARP-1 promotes AR functions [55]. Recently, two studies confirmed the association between AR, HRR and PARP, on cell lines and xenografts. They showed that androgen deprivation therapy (ADT) or enzalutamide could result in a state of BRCAness leading to sensitivity to PARP inhibition of prostate cancer cells [56, 57]. Based on these results Clarke et al*.* assessed the synergy between olaparib and abiraterone in a randomized phase II study [58]. One hundred and forty-two patients with mCRPC, who previously received docetaxel but not any NHT, were blindly randomized between abiraterone with placebo, and abiraterone with olaparib. Only 21 patients (15%) had confirmed or suspected HRR alteration (allelic status not known), 35 patients (25%) were classified as HRR wild-type, and 86 (61%) had partially characterized HRR status (HRR wild-type by plasma and/or germline test, but no valid tumor test, or no valid tumor, plasma and germline test). There was a significant improvement of rPFS in the combination arm compared to the control arm (13.8 months vs. 8.2 months, *P* = 0.034). The predefined subgroup analysis for HRR-altered patients and HRR-wild type ones was not significant, probably due to the small population. Results for OS were immature by the data cutoff, and no statistically significant differences could be seen. The toxicity of the combination was high, i.e., 54% of the patients experienced grade 3 or more adverse events in the experimental arm versus 28% in the comparator arm. Seven patients (10%), aged 66–88 years old, in the combination arm had serious cardiovascular events (4 myocardial infarctions, 1 fatal cardiac failure, 1 chronic cardiac failure, 1 fatal ischemic stroke) compared with 1 thrombotic stroke in the control arm. At baseline, 62% and 56% had cardiovascular risk factors in the combination and comparator arms, respectively. Of note, median treatment duration was longer in the combination arm (338 days vs. 253 days for abiraterone in each arm, 309 days for olaparib vs. 253 days for placebo). This study shows a synergistic interaction between NHT (abiraterone) and PARPi (olaparib), even in the absence of HRR alteration, strengthening the hypothesis that NHT induce a BRCAness state in prostate cancers. However, a relatively high cardiovascular toxicity is observed (10%) in the combination arm, leading to death in almost 3% of cases. Thus, the association should be used with caution in patients with cardiovascular history.

Based on these results, the ongoing phase III trial PROpel (NCT03732820) randomizes patients with mCRPC between abiraterone with olaparib and abiraterone with placebo, as first-line treatment, irrespective of HRR status. The primary endpoint is rPFS; secondary endpoints include OS and health-related quality of life. The effect of HRR alteration will be studied in exploratory analyses.

Several ongoing studies are evaluating the association of a PARPi combined with NHT with mainly rFPS as primary endpoint. MAGNITUDE (NCT03748641) and AMPLITUDE phase III trials (NCT04497844) randomize patients with advanced prostate cancer between abiraterone or abiraterone and niraparib in castration resistance and castration sensitive setting, respectively. The CASPAR trial (NCT04455750) is investigating a combination of enzalutamide and rucaparib compared to enzalutamide in patients with mCRPC (irrespective of HRR status). Talazoparib is being evaluated in association with enzalutamide in the TALAPRO-2 trial.

Veliparib is the PARPi with the lowest trapping activity [36] and has only been tested in combination with other treatments in mCRPC. A phase II trial aimed to assess the association of abiraterone and veliparib in patients with mCRPC irrespective of HRR status [59]. Patients were randomly assigned to receive abiraterone (arm A) or abiraterone with veliparib (300 mg twice per day, arm B). There were no statistically significant differences between the 2 arms regarding PSA response (arm A: 63.9% and arm B: 72.4%, *P* = 0.27) or median PFS (arm A: 10.1 months and arm B: 11 months, *P* = 0.99). To date, there is no other ongoing trial investigating veliparib in prostate cancer.

#### PARP inhibitors and immunotherapy

Several preclinical studies give strong rationale for using immunotherapy. Indeed, PD-L1 expression rises from localized to mCRPC [60], is higher in aggressive primary prostate cancers [61] and is more frequently expressed in dendritic cells of patients progressing under enzalutamide [62], suggesting immune escape as a progression pathway in prostate cancer. However, after a promising start and demonstration of improvements in OS in 2010 with the injection of activated autologous peripheral-blood mononuclear cells [63], the outcomes provided by immune check-point inhibitors remain mixed [64–66].

A recent study has shown that PARPi could act as immunomodulatory agents in DDR-mutated cells [67]. PARPi induce accumulation of cytosolic DNA fragments, that activate the cGAS/STING pathway, stimulating the innate immune system through an interferon-mediated response. Interferon (IFN) induces PD-L1 expression, limiting the cytotoxic immune response, which could be overcome by PD-L1 blockade. Recently, the phase II KEYNOTE-199 study, investigating pembrolizumab in patients with mCRPC refractory to docetaxel, showed that DDR deficiency could be a marker for response to immunotherapy [66]: over the 9 patients with a response and a tumor sample, 6 had an evaluable whole-exome sequencing data. Out of these 6 patients, 4 had a DDR gene alteration (1 multiple alteration including *BRCA2, FANCA,* and *RAD54* alterations; 2 with *TP53* alteration, and 1 with a *BRCA2* alteration).

Based on these results, several studies combining immunotherapy and PARPi are ongoing. The first phase II study to be published included 17 patients with mCRPC that had progressed after previous NHT, irrespective of HRR status treated by durvalumab and olaparib [68]. Nine patients (53%) had a PSA decline of more than 50%, and over the 13 RECIST-evaluable patients, 4 (30.8%) had an objective response. Of the nine patients with a PSA decline, 6 had a biallelic *BRCA2* alteration. The PSA response rate was 100% (6/6) in case of a biallelic *BRCA2* alteration compared to 27% (3/11) in the absence of a biallelic HRR alteration. Three of the 8 non-responder patients had a monoallelic *BRCA2* shallow deletion, and one had a shallow deletion of *BRCA2,* combined with a variant of uncertain significance of *BRCA2*. Grade 3–4 adverse events were comparable to the previous PARPi studies (i.e., anemia 24%, lymphopenia 12% and nausea 12%). Four patients had immune-related adverse events manageable with corticosteroids. Even if PARP inhibition was shown to increase PD-L1 expression, particularly in *BRCA2* depleted cells [69], the cohort of this preliminary study suggests that most of the efficacy is seen in the *BRCA2*-altered population and therefore may only come from olaparib with no advantage of the addition of durvalumab.

More recently, preliminary results from the phase Ib/II KEYNOTE-365 study were reported: 41 patients with heavily pretreated mCRPC (irrespective of DDR alterations) were included in cohort A, to receive olaparib and pembrolizumab at usual doses [70]. None of the patients had HRR genes alteration. The composite response rate (radiographic, or PSA or CTC response) was 15%. Twenty-one patients (51%) experienced grade 3 or more adverse events (mostly anemia, nausea and fatigue). The CRR of this study was low. It was the same as for patients without HRR alterations in the TOPARP-A study (6%), but with higher toxicity [39]. However, longer follow-up is needed.

The ongoing KEYLINK-010 study (NCT03834519) is the only phase III trial combining immunotherapy and PARPi in patients with mCRPC (unselected for HRR gene alterations), previously treated with docetaxel and one NHT. Patients are randomly assigned to receive pembrolizumab with olaparib, or investigator’s choice of NHT (abiraterone or enzalutamide, according to previous NHT).

Early results of the association of olaparib with immunotherapy are mixed. With variable toxicities, the additive or synergistic effects of such a combination, especially in HRR-altered patients, remain unclear. However, results presented are preliminary with a median follow-up under one year, as it is known that some patients can respond for a long time, as observed in a phase III clinical trial using ipilimumab [71]. Results with a larger phase III population and a longer follow-up will help us to explore the association. Other ongoing phase II trials combining immunotherapy with olaparib, niraparib or rucaparib are listed in Table 2.

#### Other combinations

Chemotherapies are tested as combination partners for PARPi. The most frequently used chemotherapies are DNA-damaging agents, such as platinum or other alkylating agents to enhance dependency on PARP enzymes, or topoisomerase inhibitors to freeze the fork and increase trapping cytotoxicity [72]. Early promising efficacy has been seen in ovarian cancers with, in return, a higher toxicity [73]. Since then, most PARPi are developed as maintenance therapies after chemotherapy. To date, the efficacy of the veliparib–temozolomide combination is the only one reported and it showed modest activity [74]. Other trials are awaited to better explore these combinations (Table 1).

Several preclinical studies have shown that the association of PARPi with other DDR inhibitors, such as ataxia telangiectasia and rad3-related kinase (ATR) inhibitors, could resensitize PARP-resistant cells or xenografts [75, 76]. Indeed, *BRCA*-deficient tumors which are resistant to PARPi seem to have an increased dependency for the ATR pathway for fork stabilization. Thus, the combination of PARPi to ATR inhibitors, unprotecting the stalled fork, may overcome the PARPi resistance and restore the synthetic lethality. The ongoing phase II study TRAP (NCT03787680) assesses the efficacy of a PARPi (olaparib) and an ATR inhibitor (AZD6738).

Based on these strong rationale, NHT, immunomodulatory agents, chemotherapies, DDR agents or other targeted therapies could free PARPi from HRR alteration dependency and enhance their efficacy. However, physicians must be aware of the potential higher toxicities.

### Biomarkers of response to PARP inhibitors

As mentioned before, not all HRR alterations have the same impact on PARPi efficacy. The TOPARP-B trial showed good CRR and ORR for *BRCA1/2* and *PALB2* altered-patients (83.3% and 52.4%, respectively, for *BRCA1/2*; 57.1% and 33.3% for *PALB2*), while almost no RECIST or biological responses were observed in *ATM* or *CDK12* altered-patients [40]. The PROfound trial confirmed these results and showed an OS improvement only in the *BRCA/ATM* group, however within this cohort *ATM* alterations did not show the same magnitude of efficacy neither in rPFS, nor in OS [10]. The same results were observed for niraparib, with an ORR of 41% in the *BRCA* group compared to 9% in the non-*BRCA* group (GALAHAD trial) [48]. TRITON-2 showed an ORR of 43.5% in the *BRCA* group, and long-lasting response for *BRIP1* and *RAD51* mutated-patients. But almost no response was observed for *ATM, CHEK2* or *CDK12*. Moreover, the ORR was lower for mono-allelic alterations (11.1%) compared to bi-allelic alterations (75.0%) [49, 50]. No differences were observed between somatic and germline *BRCA1/*2 mutations [49]. TALAPRO-1 showed a good ORR in the BRCA group and no, or few, responses for other HRR genes [52]. Finally, it is noteworthy that within the BRCA group, a recent study reported better outcomes in patients with *BRCA2* compared to *BRCA1* mutations, with no differences in terms of allelic fraction or germinal versus somatic mutations. This small retrospective study remains hypothesis -generating with, as expected, 10 times more *BRCA2* than *BRCA1* alterations [77].

Overall, these data suggest that HRR effectors, mainly *BRCA* alterations, are better than sensors of DSB for predicting PARPi efficacy (Fig. 1).

### Resistance mechanisms (Fig. 4)

**Fig. 4:**
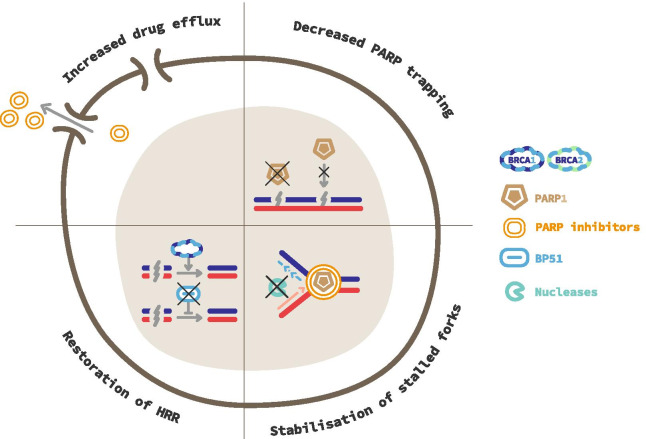
Resistance mechanism to PARP inhibitors. Increased drug efflux. Overexpression of drug-efflux transporter genes, such as *ABCB1*, increases the number of drug effluxion pumps and prevents PARP inhibitors (PARPi) from reaching cell nucleus. Decreased PARP trapping. Deletion of *PARP1* or mutations in its DNA-binding domains avoid trapping to occur. This confers cells with a resistance to PARPi. Alternatively, increased PARylation through loss of inhibitors, as PARG, produces the same effects with resistance to PARPi. Stabilization of stalled fork. Nucleases actions on nascent DNA are delayed or reduced by the inhibition of proteins in charge of their recruitment to the fork. Restoration of Homologous Recombination Repair (HRR). Mutational reversion or occurrence of a second mutation, which restores functional BRCA1/2 proteins, prevents the occurrence of synthetic lethality. Loss of inhibitors of HRR such as 53BP1 leads to the same resistance

PARPi resistance mechanisms (Fig. 4) are due to clonal selection following diverse genetic events that alter synthetic lethality. First cells can regain HRR capacity through mutational reversion of *BRCA1/BRCA2* or even more frequently due to secondary mutations that restore the open reading frame of those genes [78]. Loss of functional antagonists of BRCA1 can also restore HRR. For instance, the loss of the P53BP1 expression in BRCA1 deficient cells specifically rescues HRR [79]. *BRCA2* reversion mutations were reported on cell-free DNA in progressive patients with mCRPC treated by olaparib or talazoparib [80]. Second, the drug intracellular uptake can be reduced through up-regulation of P-glycoprotein efflux pump genes (*ABCB1*) [81]. Third, mutations of *PARP1* preventing trapping can lead to PARPi resistance, highlighting the importance of trapping in PARPi lethality [82]. Forth, loss of proteins (e.g., TET2, EZH2, PTIP, SLFN11, SMARCL1) involved directly or indirectly in the recruitment of nucleases onto unprotected *BRCA1/2* deficient replicational forks can also lead to PARPi resistance. Those data highlight that PARPi cytotoxicity in *BRCA2-* or *BRCA1-*deficient cells is also due to nuclease action on stalled replication forks by PARP [83, 84]. Finally, the microenvironment may also play a role in PARPi resistance. It has been shown that the expression of transforming growth factor beta receptor (TGFβR) kinase on malignant cells, which is activated by bone marrow stromal cells-derived transforming growth factor beta 1 (TGF-β1), could enhance the DSB repair system in leukemia cells [85]. Since bone metastases are predominant in prostate cancers it may be interesting to investigate this hypothesis, especially in *BRCA-* altered patients. Thus, a better understanding and monitoring of such resistances using liquid biopsies may guide subsequent treatments including combination of PARPi with other agents (e.g., epigenetic treatments, TGFβ inhibitors).

### PARP inhibitors compared

As seen before, four PARPi are in development for mCRPC: olaparib, niraparib, rucaparib and talazoparib. Talazoparib has the strongest trapping efficiency, and its cytotoxic efficacy is 20- to 200-fold better than the others [36, 37]. However, with olaparib, it is less selective for PARP-1 than rucaparib or niraparib [86]. Regarding efficacy as a monotherapy, the ORR between the four PARPi is roughly similar, between 40 and 50% for *BRCA*-altered patients in the four phase II studies published to date [40, 48, 49, 52]. Toxicity is also equivalent between the four molecules, mostly cytopenia, nausea and fatigue. Surprisingly, 11 patients (4%) in the olaparib group compared to 1 (1%) in the control group, experienced a pulmonary embolism in the PROfound trial [43]. This adverse event was not described in the other major studies with olaparib [7, 8, 87]. The clinical significance of the occurrence of these cases is difficult to interpret and might be due to the type of cancer or the association with ADT. Of note, all grades blood creatinine increases were seen in 10 to 15% of patients treated with olaparib, rucaparib and niraparib but not with talazoparib. This is due to the inhibition of renal transporters (MATE-1 and MATE2-K) which are involved in active secretion of creatinine for olaparib and rucaparib, and probably to hemodynamic impairment for niraparib. In most cases, there is no direct impact on renal function, and the issue is resolved with dose holds [88–90]. Regarding drug interactions, olaparib and rucaparib are metabolized by CYP450 and inducers or inhibitors of the enzymes should be avoided, while talazoparib and niraparib do not have major drug-drug interactions (accessdata.fda.gov). This last point, particularly relevant in an often older population with a substantial polypharmacy, is known to be the weakness of some NHT. The number of tablets taken daily can also be of importance, regarding observance or swallowing problems, and it ranges from one tablet for talazoparib, to 3 tablets once a day for niraparib, or 2 tablets twice a day for rucaparib and olaparib.

The evidence discussed above should be taken into account cautiously, since no direct comparisons between different PARPi have been done within one clinical trial. Perhaps, the patient’s profile as well as the development of these drugs in different indications or with different combinations will help physicians to make their choice.

### Molecular profiling, potential and limits

The use of molecular profiling is becoming increasingly prevalent in oncology. Up to now it was performed essentially on tissue samples. Mateo et al*.* profiled 470 treatment-naïve prostate biopsies, of which 61 patients also had biopsies at castrate-resistant stage [91]. The median time between the two same-patient biopsies was 45.2 months (12–211 months). An increase in AR mutations and amplifications was found, as well as increased *TP53, RB1* and *PI3K/AKT* pathway alterations in mCRPC, suggesting that they could emerge with treatment selection pressure. Conversely aberrations in HRR pathways (seen in 9 of the 61 patients) were stable between metastatic and primary biopsies, in line with previously published data [92]. The limited number of HRR-altered patients prevents from making broad conclusions, but repeating biopsies to look for late acquisition of HRR alterations may be irrelevant.

However, tissue biopsies are of insufficient quality for molecular analyses in a non-negligible quota of patients. Thus, 31% of biopsies failed molecular testing in the PROfound trial and complementary techniques are needed in order to increase the chance of screening [43]. In this last trial, plasma-derived circulating tumor DNA (ctDNA) from patients was collected as part of the screening process and was prospectively analyzed at Foundation Medicine, Inc (FMI) using the FoundationOne Liquid CDx assay. Eighty-one percent (503/619) of ctDNA samples tested yielded a result. High concordance between tumor tissues and ctDNA was found, with 81% positive percentage agreement and 92% negative percentage agreement [93]. Of the 181 patients in the *BRCA/ATM* cohort of the study who consented and provided a plasma sample for ctDNA testing, 42 (23.2%) samples failed analyses. *BRCA/ATM* alterations were identified in 111/139 patients (79.9%). The rPFS [HR = 0.33 (95% CI 0.21–0.53)] improvement was in the same order of magnitude as the one in the Intention to Treat population identified by tumor tissue testing [HR = 0.34 (95% CI 0.25—0.47)] [94]. Thus, ctDNA could offer additional opportunities to patients who are not eligible for tumor tissue testing. However, not all patients have concordant results and it can be tough to estimate allelic fraction. Indeed, it depends on tumor cell percentage, clonality and ploidy of the tumor sample. To enhance efficiency, ctDNA analyses should be done before the introduction of new treatment, when the disease is progressing, especially since ctDNA availability reduces right after the initiation of an effective treatment, such as ADT [95]. The optimal approach for biomarkers should combine, if possible, tissue and liquid biopsies as they can be complementary.

## PARP inhibitors in 2021: Where are we? What will be the future challenges?

PARP inhibitors are paving the way of precision medicine in prostate cancer, followed by drugs targeting the PI3k AKT mTOR pathway. While roughly one quarter of patients with mCRPC harbor somatic or genetic HRR gene mutations, not all derive benefit from PARPi. Olaparib obtained FDA approval at a dose of 300 mg twice daily on all genes tested (except *PP2R2A*), based on the first results of the PROfound trial with improvement on rPFS. However, given the benefit in OS for cohort A, EMEA approval has just been formalized, and restricted to *BRCA1/2* regardless of the somatic or germinal status (*ATM* was excluded). The benefit in OS for cohort A may be nuanced by a “weak” standard arm (i.e., no chemotherapy and half of patients with 2 back-to-back NHT as first lines for mCRPC). The PROfound trial has been designed before the results of the CARD study which highlighted the necessity for eligible patients not to delay chemotherapy in order to maintain a survival benefit [47]. Results of the TRITON-3 trial (NCT02975934; Table 1) which incorporates docetaxel in the standard arm will be of interest. It is noteworthy that results of the TOPARP-B trial suggest a better ORR but with higher toxicity using 400 mg twice daily compared to 300 mg twice daily [40]. That said, these data might be interesting for dosing patients with good tolerance and absence of response. Olaparib also improved time to pain progression and HRQoL, strengthening its clinical impact [44, 45]. These patient-reported outcomes are taken into consideration for EMEA and FDA approvals in the setting of mCRPC. Treatment with PARPi used alone is based on a screening of genes, with questions regarding the panel of genes to test, accessibility, cost and the population who may benefit from such a test. While HRR-mutated genes seem to occur early, with a suggested low enrichment from localized to metastatic disease [91, 92], the feasibility of large screening programs with archived biopsy samples may be challenging, as well as new biopsies on osteoblastic bone metastases, which are the only lesions accessible for roughly half of patients with mCRPC. For instance, in the PROfound trial screening, 30% of biopsies were not suitable for DNA analysis [96]. High concordance between tumor tissues and ctDNA was found, with 81% positive percentage agreement and 92% negative percentage agreement [93]. Liquid biopsies may then be helpful. However, some limitations regarding the determination of the allelic fraction results can be seen. Detection of somatic HRR-mutated genes first may be more efficient, since they are two times as frequent as germinal mutations. Nevertheless, in cases of somatic mutation, a germinal mutation must be sought for genetic counseling. Issues related to patient consent to involve their family in their research of genetic patrimony may be challenging and raise ethical considerations. It is then of importance in each country, to think about a specific patient’s pathway related to a biomarker-based approach (depending on biopsies suitable for analysis), and potentially involving genetic counseling. Allelic distribution seems to be important to better predict efficacy of PARPi, since only a biallelic silencing is associated with loss of function of tumor suppressor genes. However, to date its real impact in daily clinical practice is still unknown despite promising results for ORR [48, 49]. Prospective and retrospective studies investigating HRR status [33, 96] in patients with mCRPC, showed co-occurrence between 2 or more genes involved (mostly with *BRCA2, CDK12* and *ATM*) in this pathway, raising questions about biological impact on HRR and their predictive nature. Combination studies using PARPi with either NHT or immune checkpoint inhibitors based on robust rationale are still under evaluation with ongoing phase III trials. While benefits may be maintained regardless of HRR status, major toxicities have to be prevented in combination with NHT. Finally, platinum may be efficient in this setting since they induce intra-stand cross-links repairs using NER and HRR systems. A recent real-world cohort study investigating carboplatin and olaparib in patients with mCRPC and mutations in either *BRCA1, BRCA2* and *ATM* did not find any differences in PFS between the 2 drugs [97]. While PARPi is indicated according to platinum sensitivity in ovarian and pancreatic cancers, data in prostate cancer need further investigation to better delineate the role of platinum, with potential leads following resistance to PARPi (NCT04288687, PLATI-PARP trial).

## Conclusions

Prostate cancer is the first disease where overall survival has been improved using a PARP inhibitor. Up until now, similarly to ovarian cancers [7], somatic and/or germinal *BRCA 1* and *2* aberrations seem to be the most predictive biomarkers of efficacy of PARPi. Data regarding the other HRR genes are generating hypotheses for further studies, though some challenges must still be overcome regarding the screening of these patients, for instance involving genetic counseling. Results of other ongoing phase III trials assessing the efficacy of a PARPi used alone or in combination are awaited, to better define their place with regard to standard treatments and platinum-based chemotherapies.

## Data Availability

Not applicable.

## Abbreviations

ADT: Androgen deprivation therapy
AR: Androgen receptor
ATR: Ataxia telangiectasia and rad3-related kinase
BER: Base excision repair
CRR: Composite response rate
CSS: Cause-specific survival
CTC: Circulating tumor cell
ctDNA: Circulating tumor DNA
DDR: DNA-damage response
FANC: Fanconi Anemia
FMI: Foundation Medicine, Inc
HR: Hazard ratio
HRD: Homologous recombination deficient
HRQoL: Health-related quality of life
HRR: Homologous recombination repair
IFN: Interferon
LOH: Loss of heterozygosity
mCRPC: Metastatic castration-resistant prostate cancer
MMEJ: Microhomology-mediated end joining
MMR: Mismatch repair
NAD +: Nicotinamide adenine dinucleotide
NER: Nucleotide excision repair
NGS: Next-generation sequencing
NHEJ: Non-homologous end joining
NHT: New-hormonal therapy
ORR: Objective response rate
OS: Overall survival
PARP: Poly(adenosine diphosphate-ribose) polymerase
PARPi: Poly(adenosine diphosphate-ribose) polymerase inhibitor
PFS: Progression-free survival
RECIST: Response evaluation criteria in solid tumors
rPFS: Radiographic progression-free survival
SSB: Single-strand break
TGF-β1: Transforming growth factor beta 1
TGFβR: Transforming growth factor beta receptor

## References

[1] Bray F, Ferlay J, Soerjomataram I, Siegel RL, Torre LA, Jemal A (2018). Global cancer statistics 2018: GLOBOCAN estimates of incidence and mortality worldwide for 36 cancers in 185 countries. CA Cancer J Clin.

[2] Parker C, Castro E, Fizazi K, Heidenreich A, Ost P, Procopio G (2020). Prostate cancer: ESMO clinical practice guidelines for diagnosis, treatment and follow-up. Ann Oncol.

[3] Tan ME, Li J, Xu HE, Melcher K, Yong E (2015). Androgen receptor: structure, role in prostate cancer and drug discovery. Acta Pharmacol Sin.

[4] Robinson D, Van Allen EM, Wu Y-M, Schultz N, Lonigro RJ, Mosquera J-M (2015). Integrative clinical genomics of advanced prostate cancer. Cell.

[5] Abida W, Armenia J, Gopalan A, Brennan R, Walsh M, Barron D, et al. Prospective genomic profiling of prostate cancer across disease states reveals germline and somatic alterations that may affect clinical decision making. JCO Precis Oncol. 2017. 10.1200/PO.17.00029 PMC5558263 28825054

[6] Castro E, Goh C, Olmos D, Saunders E, Leongamornlert D, Tymrakiewicz M (2013). Germline BRCA mutations are associated with higher risk of nodal involvement, distant metastasis, and poor survival outcomes in prostate cancer. J Clin Oncol.

[7] Moore K, Colombo N, Scambia G, Kim B-G, Oaknin A, Friedlander M (2018). Maintenance olaparib in patients with newly diagnosed advanced ovarian cancer. N Engl J Med.

[8] Golan T, Hammel P, Reni M, Van Cutsem E, Macarulla T, Hall MJ (2019). Maintenance olaparib for germline BRCA-mutated metastatic pancreatic cancer. N Engl J Med..

[9] Litton JK, Rugo HS, Ettl J, Hurvitz SA, Gonçalves A, Lee K-H (2018). Talazoparib in patients with advanced breast cancer and a germline BRCA mutation. N Engl J Med.

[10] Hussain M, Mateo J, Fizazi K, Saad F, Shore N, Sandhu S (2020). Survival with olaparib in metastatic castration-resistant prostate cancer. N Engl J Med.

[11] Loeb LA (1991). Mutator phenotype may be required for multistage carcinogenesis. Cancer Res.

[12] Otto H, Reche PA, Bazan F, Dittmar K, Haag F, Koch-Nolte F (2005). In silico characterization of the family of PARP-like poly(ADP-ribosyl)transferases (pARTs). BMC Genom.

[13] Juarez-Salinas H, Levi V, Jacobson EL, Jacobson MK (1982). Poly(ADP-ribose) has a branched structure in vivo. J Biol Chem.

[14] Okano S, Lan L, Caldecott KW, Mori T, Yasui A (2003). Spatial and temporal cellular responses to single-strand breaks in human cells. Mol Cell Biol.

[15] Pines A, Vrouwe MG, Marteijn JA, Typas D, Luijsterburg MS, Cansoy M (2012). PARP1 promotes nucleotide excision repair through DDB2 stabilization and recruitment of ALC1. J Cell Biol.

[16] Wang ZQ, Stingl L, Morrison C, Jantsch M, Los M, Schulze-Osthoff K (1997). PARP is important for genomic stability but dispensable in apoptosis. Genes Dev.

[17] Liang F, Han M, Romanienko PJ, Jasin M (1998). Homology-directed repair is a major double-strand break repair pathway in mammalian cells. Proc Natl Acad Sci.

[18] Stolz A, Ertych N, Bastians H (2011). Tumor suppressor CHK2: regulator of DNA damage response and mediator of chromosomal stability. Clin Cancer Res Off J Am Assoc Cancer Res.

[19] Zhang J, Willers H, Feng Z, Ghosh JC, Kim S, Weaver DT (2004). Chk2 phosphorylation of BRCA1 regulates DNA double-strand break repair. Mol Cell Biol.

[20] Zhang F, Fan Q, Ren K, Andreassen PR (2009). PALB2 functionally connects the breast cancer susceptibility proteins BRCA1 and BRCA2. Mol Cancer Res MCR.

[21] Liu J, Doty T, Gibson B, Heyer W-D (2010). Human BRCA2 protein promotes RAD51 filament formation on RPA-covered single-stranded DNA. Nat Struct Mol Biol.

[22] Grompe M, D’Andrea A (2001). Fanconi anemia and DNA repair. Hum Mol Genet.

[23] Maxwell KN, Wubbenhorst B, Wenz BM, De Sloover D, Pluta J, Emery L (2017). BRCA locus-specific loss of heterozygosity in germline BRCA1 and BRCA2 carriers. Nat Commun.

[24] Pritchard CC, Mateo J, Walsh MF, De Sarkar N, Abida W, Beltran H (2016). Inherited DNA-repair gene mutations in men with metastatic prostate cancer. N Engl J Med.

[25] Na R, Zheng SL, Han M, Yu H, Jiang D, Shah S (2017). Germline mutations in ATM and BRCA1/2 distinguish risk for lethal and indolent prostate cancer and are associated with early age at death. Eur Urol.

[26] Edwards SM, Evans DGR, Hope Q, Norman AR, Barbachano Y, Bullock S (2010). Prostate cancer in BRCA2 germline mutation carriers is associated with poorer prognosis. Br J Cancer.

[27] Alexandrov LB, Nik-Zainal S, Wedge DC, Aparicio SAJR, Behjati S, Biankin AV (2013). Signatures of mutational processes in human cancer. Nature.

[28] Wu Y, Yu H, Zheng SL, Na R, Mamawala M, Landis T (2018). A comprehensive evaluation of CHEK2 germline mutations in men with prostate cancer. Prostate.

[29] Page EC, Bancroft EK, Brook MN, Assel M, Hassan Al Battat M, Thomas S (2019). Interim results from the IMPACT study: evidence for prostate-specific antigen screening in BRCA2 mutation carriers. Eur Urol.

[30] Nicolosi P, Ledet E, Yang S, Michalski S, Freschi B, O’Leary E (2019). Prevalence of germline variants in prostate cancer and implications for current genetic testing guidelines. JAMA Oncol.

[31] Antonarakis ES, Isaacsson Velho P, Fu W, Wang H, Agarwal N, Sacristan Santos V (2020). CDK12-altered prostate cancer: clinical features and therapeutic outcomes to standard systemic therapies, poly (ADP-Ribose) polymerase inhibitors, and PD-1 inhibitors. JCO Precis Oncol.

[32] Schweizer MT, Ha G, Gulati R, Brown LC, McKay RR, Dorff T (2020). CDK12-mutated prostate cancer: clinical outcomes with standard therapies and immune checkpoint blockade. JCO Precis Oncol..

[33] Rescigno P, Gurel B, Pereira R, Crespo M, Rekowski J, Rediti M, et al. Characterizing CDK12-mutated prostate cancers. Clin Cancer Res. 2020;1078–0432.CCR-20–2371. 10.1158/1078-0432.CCR-20-2371 PMC7855716 32988971

[34] Castro E, Romero-Laorden N, Del Pozo A, Lozano R, Medina A, Puente J (2019). PROREPAIR-B: a prospective cohort study of the impact of germline DNA repair mutations on the outcomes of patients with metastatic castration-resistant prostate cancer. J Clin Oncol Off J Am Soc Clin Oncol.

[35] Antonarakis ES, Lu C, Luber B, Liang C, Wang H, Chen Y (2018). Germline DNA-repair gene mutations and outcomes in men with metastatic castration-resistant prostate cancer receiving first-line abiraterone and enzalutamide. Eur Urol.

[36] Murai J, Huang SN, Das BB, Renaud A, Zhang Y, Doroshow JH (2012). Trapping of PARP1 and PARP2 by clinical PARP inhibitors. Cancer Res..

[37] Murai J, Huang S-YN, Renaud A, Zhang Y, Ji J, Takeda S (2014). Stereospecific PARP trapping by BMN 673 and comparison with olaparib and rucaparib. Mol Cancer Ther.

[38] Mijic S, Zellweger R, Chappidi N, Berti M, Jacobs K, Mutreja K (2017). Replication fork reversal triggers fork degradation in BRCA2-defective cells. Nat Commun.

[39] Mateo J, Carreira S, Sandhu S, Miranda S, Mossop H, Perez-Lopez R (2015). DNA-repair defects and olaparib in metastatic prostate cancer. N Engl J Med.

[40] Mateo J, Porta N, Bianchini D, McGovern U, Elliott T, Jones R (2020). Olaparib in patients with metastatic castration-resistant prostate cancer with DNA repair gene aberrations (TOPARP-B): a multicentre, open-label, randomised, phase 2 trial. Lancet Oncol.

[41] Tutt A, Robson M, Garber JE, Domchek SM, Audeh MW, Weitzel JN (2010). Oral poly(ADP-ribose) polymerase inhibitor olaparib in patients with BRCA1 or BRCA2 mutations and advanced breast cancer: a proof-of-concept trial. Lancet Lond Engl.

[42] Audeh MW, Carmichael J, Penson RT, Friedlander M, Powell B, Bell-McGuinn KM (2010). Oral poly(ADP-ribose) polymerase inhibitor olaparib in patients with BRCA1 or BRCA2 mutations and recurrent ovarian cancer: a proof-of-concept trial. Lancet Lond Engl.

[43] de Bono J, Mateo J, Fizazi K, Saad F, Shore N, Sandhu S (2020). Olaparib for metastatic castration-resistant prostate cancer. N Engl J Med..

[44] Saad F, Roubaud G, Procopio G, Shore ND, Fizazi K, Thiery-Vuillemin A (2020). Impact of olaparib vs physician’s choice of new hormonal agent (pcNHA) on burden of pain in metastatic castration-resistant prostate cancer (mCRPC): PROfound. J Clin Oncol..

[45] Thiery-Vuillemin A, De Bono JS, Saad F, Procopio G, Shore ND, Fizazi K (2020). Health-related quality of life (HRQoL) for olaparib versus enzalutamide or abiraterone in metastatic castration-resistant prostate cancer (mCRPC) with homologous recombination repair (HRR) gene alterations: PROfound. J Clin Oncol..

[46] Oh WK, Cheng WY, Miao R, Vekeman F, Gauthier-Loiselle M, Duh MS (2018). Real-world outcomes in patients with metastatic castration-resistant prostate cancer receiving second-line chemotherapy versus an alternative androgen receptor-targeted agent (ARTA) following early progression on a first-line ARTA in a US community oncology setting. Urol Oncol.

[47] de Wit R, de Bono J, Sternberg CN, Fizazi K, Tombal B, Wülfing C (2019). Cabazitaxel versus abiraterone or enzalutamide in metastatic prostate cancer. N Engl J Med.

[48] Smith MR, Sandhu SK, Kelly WK, Scher HI, Efstathiou E, Lara PN (2019). Pre-specified interim analysis of GALAHAD: a phase II study of niraparib in patients (pts) with metastatic castration-resistant prostate cancer (mCRPC) and biallelic DNA-repair gene defects (DRD). Ann Oncol.

[49] Abida W, Patnaik A, Campbell D, Shapiro J, Bryce AH, McDermott R (2020). Rucaparib in men with metastatic castration-resistant prostate cancer harboring a BRCA1 or BRCA2 gene alteration. J Clin Oncol.

[50] Abida W, Campbell D, Patnaik A, Shapiro JD, Sautois B, Vogelzang NJ (2020). Non-BRCA DNA damage repair gene alterations and response to the PARP inhibitor rucaparib in metastatic castration-resistant prostate cancer: analysis from the phase II TRITON2 study. Clin Cancer Res.

[51] Shen Y, Rehman FL, Feng Y, Boshuizen J, Bajrami I, Elliott R (2013). BMN 673, a novel and highly potent PARP1/2 inhibitor for the treatment of human cancers with DNA repair deficiency. Clin Cancer Res Off J Am Assoc Cancer Res.

[52] De Bono JS, Mehra N, Higano CS, Saad F, Buttigliero C, van Oort IM (2020). TALAPRO-1: phase II study of talazoparib (TALA) in patients (pts) with DNA damage repair alterations (DDRm) and metastatic castration-resistant prostate cancer (mCRPC)—updated interim analysis (IA). J Clin Oncol.

[53] De Bono JS, Mehra N, Higano CS, Saad F, Buttigliero C, van Oort IM (2021). TALAPRO-1: phase II study of talazoparib (TALA) in patients (pts) with DNA damage repair alterations (DDRm) and metastatic castration-resistant prostate cancer (mCRPC). J Clin Oncol.

[54] Polkinghorn WR, Parker JS, Lee MX, Kass EM, Spratt DE, Iaquinta PJ (2013). Androgen receptor signaling regulates DNA repair in prostate cancers. Cancer Discov.

[55] Schiewer MJ, Goodwin JF, Han S, Brenner JC, Augello MA, Dean JL (2012). Dual roles of PARP-1 promote cancer growth and progression. Cancer Discov.

[56] Asim M, Tarish F, Zecchini HI, Sanjiv K, Gelali E, Massie CE (2017). Synthetic lethality between androgen receptor signalling and the PARP pathway in prostate cancer. Nat Commun..

[57] Li L, Karanika S, Yang G, Wang J, Park S, Broom BM (2017). Androgen receptor inhibitor-induced “BRCAness” and PARP inhibition are synthetically lethal for castration-resistant prostate cancer. Sci Signal..

[58] Clarke N, Wiechno P, Alekseev B, Sala N, Jones R, Kocak I (2018). Olaparib combined with abiraterone in patients with metastatic castration-resistant prostate cancer: a randomised, double-blind, placebo-controlled, phase 2 trial. Lancet Oncol.

[59] Hussain M, Daignault-Newton S, Twardowski PW, Albany C, Stein MN, Kunju LP (2018). Targeting androgen receptor and DNA repair in metastatic castration-resistant prostate cancer: results from NCI 9012. J Clin Oncol Off J Am Soc Clin Oncol.

[60] Haffner MC, Guner G, Taheri D, Netto GJ, Palsgrove DN, Zheng Q (2018). Comprehensive evaluation of programmed death-ligand 1 expression in primary and metastatic prostate cancer. Am J Pathol.

[61] Gevensleben H, Dietrich D, Golletz C, Steiner S, Jung M, Thiesler T (2016). The immune checkpoint regulator PD-L1 is highly expressed in aggressive primary prostate cancer. Clin Cancer Res.

[62] Bishop JL, Sio A, Angeles A, Roberts ME, Azad AA, Chi KN (2014). PD-L1 is highly expressed in Enzalutamide resistant prostate cancer. Oncotarget Impact J.

[63] Kantoff PW, Higano CS, Shore ND, Berger ER, Small EJ, Penson DF (2010). Sipuleucel-T immunotherapy for castration-resistant prostate cancer. N Engl J Med.

[64] Kwon ED, Drake CG, Scher HI, Fizazi K, Bossi A, van den Eertwegh AJM (2014). Ipilimumab versus placebo after radiotherapy in patients with metastatic castration-resistant prostate cancer that had progressed after docetaxel chemotherapy (CA184-043): a multicentre, randomised, double-blind, phase 3 trial. Lancet Oncol.

[65] Beer TM, Kwon ED, Drake CG, Fizazi K, Logothetis C, Gravis G (2016). Randomized, double-blind, phase III Trial of ipilimumab versus placebo in asymptomatic or minimally symptomatic patients with metastatic chemotherapy-naive castration-resistant prostate cancer. J Clin Oncol.

[66] Antonarakis ES, Piulats JM, Gross-Goupil M, Goh J, Ojamaa K, Hoimes CJ (2020). Pembrolizumab for treatment-refractory metastatic castration-resistant prostate cancer: multicohort, open-label phase II KEYNOTE-199 study. J Clin Oncol Off J Am Soc Clin Oncol.

[67] Chabanon RM, Muirhead G, Krastev DB, Adam J, Morel D, Garrido M (2019). PARP inhibition enhances tumor cell–intrinsic immunity in ERCC1-deficient non-small cell lung cancer. J Clin Invest..

[68] Karzai F, VanderWeele D, Madan RA, Owens H, Cordes LM, Hankin A (2018). Activity of durvalumab plus olaparib in metastatic castration-resistant prostate cancer in men with and without DNA damage repair mutations. J Immunother Cancer.

[69] Sato H, Niimi A, Yasuhara T, Permata TBM, Hagiwara Y, Isono M (2017). DNA double-strand break repair pathway regulates PD-L1 expression in cancer cells. Nat Commun..

[70] Yu EY, Massard C, Retz M, Tafreshi A, Carles Galceran J, Hammerer P (2019). Keynote-365 Cohort a: pembrolizumab (pembro) plus olaparib in docetaxel-pretreated patients (pts) with metastatic castrate-resistant prostate cancer (mCRPC). J Clin Oncol..

[71] Fizazi K, Drake CG, Beer TM, Kwon ED, Scher HI, Gerritsen WR (2020). Final analysis of the ipilimumab versus placebo following radiotherapy phase III trial in postdocetaxel metastatic castration-resistant prostate cancer identifies an excess of long-term survivors. Eur Urol.

[72] Murai J, Zhang Y, Morris J, Ji J, Takeda S, Doroshow JH (2014). Rationale for poly(ADP-ribose) polymerase (PARP) inhibitors in combination therapy with camptothecins or temozolomide based on PARP trapping versus catalytic inhibition. J Pharmacol Exp Ther.

[73] Oza AM, Cibula D, Benzaquen AO, Poole C, Mathijssen RHJ, Sonke GS (2015). Olaparib combined with chemotherapy for recurrent platinum-sensitive ovarian cancer: a randomised phase 2 trial. Lancet Oncol.

[74] Hussain M, Carducci MA, Slovin S, Cetnar J, Qian J, McKeegan EM (2014). Targeting DNA repair with combination veliparib (ABT-888) and temozolomide in patients with metastatic castration-resistant prostate cancer. Invest New Drugs.

[75] Yazinski SA, Comaills V, Buisson R, Genois M-M, Nguyen HD, Ho CK (2017). ATR inhibition disrupts rewired homologous recombination and fork protection pathways in PARP inhibitor-resistant BRCA-deficient cancer cells. Genes Dev.

[76] Kim H, Xu H, George E, Hallberg D, Kumar S, Jagannathan V (2020). Combining PARP with ATR inhibition overcomes PARP inhibitor and platinum resistance in ovarian cancer models. Nat Commun..

[77] Taza F, Holler AE, Adra N, Ashkar R, Sokolova A, Kessel A (2021). Differential activity of PARP inhibitors in BRCA1- versus BRCA2-altered metastatic castration-resistant prostate cancer (mCRPC). J Clin Oncol.

[78] Edwards SL, Brough R, Lord CJ, Natrajan R, Vatcheva R, Levine DA (2008). Resistance to therapy caused by intragenic deletion in BRCA2. Nature.

[79] Bunting SF, Callen E, Kozak ML, Kim J-M, Wong N, Lopez-Contreras AJ (2012). BRCA1 functions independently of homologous recombination in DNA interstrand cross-link repair. Mol Cell.

[80] Quigley D, Alumkal JJ, Wyatt AW, Kothari V, Foye A, Lloyd P (2017). Analysis of circulating cell-free DNA identifies multiclonal heterogeneity of BRCA2 reversion mutations associated with resistance to PARP inhibitors. Cancer Discov.

[81] Rottenberg S, Jaspers JE, Kersbergen A, van der Burg E, Nygren AOH, Zander SAL (2008). High sensitivity of BRCA1-deficient mammary tumors to the PARP inhibitor AZD2281 alone and in combination with platinum drugs. Proc Natl Acad Sci U S A.

[82] Pettitt SJ, Krastev DB, Brandsma I, Dréan A, Song F, Aleksandrov R (2018). Genome-wide and high-density CRISPR-Cas9 screens identify point mutations in PARP1 causing PARP inhibitor resistance. Nat Commun.

[83] Chaudhuri AR, Callen E, Ding X, Gogola E, Duarte AA, Lee J-E (2016). Replication fork stability confers chemoresistance in BRCA-deficient cells. Nature.

[84] Kharat SS, Ding X, Swaminathan D, Suresh A, Singh M, Sengodan SK, et al. Degradation of 5hmC-marked stalled replication forks by APE1 causes genomic instability. Sci Signal [Internet]. American Association for the Advancement of Science; 2020 [cited 2021 Jan 28];13. https://stke-sciencemag-org.proxy.insermbiblio.inist.fr/content/13/645/eaba8091 10.1126/scisignal.aba8091 PMC7575062 32817374

[85] Le BV, Podszywalow-Bartnicka P, Maifrede S, Sullivan-Reed K, Nieborowska-Skorska M, Golovine K (2020). TGFβR-SMAD3 signaling induces resistance to PARP inhibitors in the bone marrow microenvironment. Cell Rep.

[86] Thorsell A-G, Ekblad T, Karlberg T, Löw M, Pinto AF, Trésaugues L (2017). Structural basis for potency and promiscuity in poly(ADP-ribose) polymerase (PARP) and Tankyrase inhibitors. J Med Chem.

[87] Robson M, Im S-A, Senkus E, Xu B, Domchek SM, Masuda N (2017). Olaparib for metastatic breast cancer in patients with a germline BRCA mutation. N Engl J Med.

[88] Liao M, Jaw-Tsai S, Beltman J, Simmons AD, Harding TC, Xiao JJ (2020). Evaluation of in vitro absorption, distribution, metabolism, and excretion and assessment of drug–drug interaction of rucaparib, an orally potent poly(ADP-ribose) polymerase inhibitor. Xenobiotica.

[89] McCormick A, Swaisland H (2017). In vitro assessment of the roles of drug transporters in the disposition and drug–drug interaction potential of olaparib. Xenobiotica.

[90] Lazareth H, Delanoy N, Cohen R, Boissier E, Ayari H, Combe P (2020). Nephrotoxicity associated with Niraparib. Am J Kidney Dis.

[91] Mateo J, Seed G, Bertan C, Rescigno P, Dolling D, Figueiredo I (2020). Genomics of lethal prostate cancer at diagnosis and castration resistance. J Clin Invest..

[92] Wedge DC, Gundem G, Mitchell T, Woodcock DJ, Martincorena I, Ghori M (2018). Sequencing of prostate cancers identifies new cancer genes, routes of progression and drug targets. Nat Genet.

[93] Chi KN, Barnicle A, Sibilla C, Lai Z, Corcoran C, Williams JA (2021). Concordance of BRCA1, BRCA2 (BRCA), and ATM mutations identified in matched tumor tissue and circulating tumor DNA (ctDNA) in men with metastatic castration-resistant prostate cancer (mCRPC) screened in the PROfound study. J Clin Oncol.

[94] Matsubara N, De Bono JS, Olmos D, Procopio G, Kawakami S, Urun Y (2021). Olaparib efficacy in patients with metastatic castration-resistant prostate cancer (mCRPC) carrying circulating tumor (ct) DNA alterations in BRCA1, BRCA2 or ATM: Results from the PROfound study. J Clin Oncol.

[95] Vandekerkhove G, Struss WJ, Annala M, Kallio HML, Khalaf D, Warner EW (2019). Circulating tumor DNA abundance and potential utility in de novo metastatic prostate cancer. Eur Urol.

[96] De Bono JS, Fizazi K, Saad F, Shore N, Sandhu SK, Mehra N (2019). Central, prospective detection of homologous recombination repair gene mutations (HRRm) in tumour tissue from >4000 men with metastatic castration-resistant prostate cancer (mCRPC) screened for the PROfound study. Ann Oncol..

[97] Berchuck JE, Silver R, Bakouny Z, Abou Alaiwi S, Hamid A, Sweeney C (2020). Response to olaparib or carboplatin in a real-world cohort of men with DNA damage repair (DDR) deficient metastatic castration-resistant prostate cancer (mCRPC). J Clin Oncol..

